# Metabolic consequences of discretionary fortified beverage consumption containing excessive vitamin B levels in adolescents

**DOI:** 10.1371/journal.pone.0209913

**Published:** 2019-01-17

**Authors:** Shyamchand Mayengbam, Heidi Virtanen, Dustin S. Hittel, Charlene Elliott, Raylene A. Reimer, Hans J. Vogel, Jane Shearer

**Affiliations:** 1 Faculty of Kinesiology, University of Calgary, Calgary, Alberta, Canada; 2 Alberta Children’s Hospital Research Institute, Alberta Children’s Hospital, Calgary, Alberta, Canada; 3 Department of Pediatrics, Alberta Children’s Hospital, Calgary, Alberta, Canada; 4 Department of Biochemistry and Molecular Biology, Cumming School of Medicine, University of Calgary, Alberta, Canada; 5 Department of Communication, Media, and Film, Faculty of Arts, University of Calgary, Calgary, Alberta, Canada; 6 Department of Biological Sciences, University of Calgary, Calgary, Alberta, Canada; Vanderbilt University, UNITED STATES

## Abstract

Over the past decade, there has been a substantial increase in the number of beverage products containing added vitamins and minerals. Often viewed as a healthier choice by consumers, the metabolic impacts of excessive vitamin consumption are relatively unknown, especially in children. The aim of this study was to examine the effects of a widely available, vitamin fortified beverage (5h Energy Decaffeinated) on insulin sensitivity, metabolic hormones and serum metabolomic responses in adolescents. Twenty adolescents (13-19y, 10M/10F) completed two randomized trials, consuming either coloured water as placebo (PL) or a vitamin fortified, sugar free beverage (FB, 1.5ml/kg) 40min prior to a modified oral glucose tolerance test (OGTT, 1.75g/kg glucose). Samples were collected at baseline and at 30, 45, 60, 90 and 120min during the OGTT. No differences in blood glucose response were observed between the treatments. However, compared to PL, postprandial plasma C-peptide and insulin excursion was significantly greater with FB, resulting in a 28% decline in the insulin sensitivity index. This was accompanied by elevated GLP-1, glucagon and PYY responses with FB compared to PL. Serum metabolomics (^1^H-NMR) analysis also revealed perturbations to vitamin B-linked one carbon metabolism flux with FB consumption that became more pronounced over time. These included a transient reduction in homocysteine flux accompanied by increases in betaine, vitamin B6, vitamin B12, choline, folate and taurine. Although these impacts are likely short-lived, results show that beverages fortified with excessive amounts of vitamins are not metabolically inert, but likely result in greater insulin secretion, differential gut hormone secretion and elevated one-carbon flux to process the excessive vitamin loads.

## Introduction

Discretionary fortification refers to the addition of micronutrients such as vitamins and minerals to foods and beverages by manufacturers. In recent years, the sales of discretionary fortified beverages (FB), including vitamin waters, juices and energy drinks have flourished with a 638% increase in sales over the past 12 years [1, 2]. On average, these novel beverages contain 4 to 5 micronutrients, often in quantities well in excess of the Estimated Average Requirements (EAR) [3]. In fact, 83% of recently analyzed beverages in this category contained at least 1 nutrient exceeding EAR for children and most contained 3 or more nutrients in excess [4].

Although advertised as a convenient way for consumers to meet their daily micronutrient requirements and improve health [5], concerns have been raised over the growing consumption of fortified foods and beverages, especially in pediatric populations [6]. While there is limited data on how beverage fortification has altered micronutrient intakes, it is clear that there has been a sharp increase in consumption with 8.5% of U.S. adolescents consuming FB weekly, with increased prevalence among older teens [7–9]. Expectedly, increased consumption has also been accompanied by an elevated caloric intake from fortified sources in the past decade [10]. While most fortifications pose no harm for consumers, a subcategory of FB with excessive vitamin levels has emerged. Such products are often marketed to, and consumed by adolescents [4].

Given this, the purpose of the present study was to examine the impacts of FB consumption, containing excessive levels of B-vitamins, on glucose metabolism, incretin responses and metabolomics profiles in adolescents. In particular, we were interested in how FB consumption affected responsiveness to a subsequent glucose load. Secondary aims were to examine the metabolic impacts of excessive B-vitamins, common FB additives, employing a proton nuclear magnetic resonance spectroscopy-based metabolomics (^1^H-NMR) approach. As individual responses to vitamin B metabolism are known to be impacted by genetics [11, 12], a precursory examination of gene-metabolite interactions using common B-vitamin linked variants (single nucleotide polymorphisms, SNPs) was also conducted.

## Materials and methods

### Subjects and study design

This study was approved by the Conjoint Health Research Ethics Board at the University of Calgary (REB14-1093) and registered at ClinicalTrials.gov (NCT03512496). Twenty healthy adolescents (10 male, 10 female) aged 13–19 years were recruited from the community (Alberta, Canada). Informed consents (child assent and parent consent) were obtained and basic demographic information, anthropometric measures including height, weight, waist circumference and body fat was collected. Self-report tanner staging was assessed as described [13, 14]. Menstrual phase was recorded, but not controlled in female participants.

In this randomized, double-blind, cross-over trial, participants completed two trials, placebo (PL, coloured water) and fortified beverage (FB, 5 Hour Energy^™^ Decaffeinated), separated by at least 1 week. The composition of FB is shown in the S1 Table. FB was administered based on body mass (1.5ml/kg) and equivalent to a 13-year-old (~38kg) consuming one bottle (57ml) of the beverage [15].

On the day of the trial, participants arrived at the laboratory following an overnight fast. An indwelling catheter was placed in the antecubital vein by a nurse to facilitate repeated blood sampling. Following baseline blood sampling, subjects consumed the test beverages and rested for 40min before undergoing a modified oral glucose tolerance test (OGTT, 1.75g/kg to a maximum of 75g, Trutol, Thermo Fisher, USA). Additional blood samples were collected at 0, 30, 45, 60, 90 and 120min. A study schematic of design and time points of blood collection is provided in S1 Fig.

### Laboratory analyses

Blood samples collected at each time point were centrifuged and serum aliquots were frozen at -80°C for subsequent analyses. For metabolic hormones, blood was drawn into cooled EDTA vacutainer containing inhibitors: diprotinin-A (0.034g/L; MP Biomedicals), protease inhibitor (1g/L; Sigma Aldrich), and Roche Pefabloc (1g/L). Plasma glucose concentrations were analyzed by the use of a colorimetric-based glucose oxidase assay (Cayman Chemical, USA). A total of nine metabolic hormone biomarkers were simultaneously quantified via Human Metabolic Array 9-Plex (Eve Technologies, Canada) using the Bio-Plex 200 system (Bio-Rad, USA) according to protocol. The 9-Plex consisted of C-Peptide, ghrelin (active), glucose-dependent insulinotropic peptide (GIP), glucagon-like peptide-1 (GLP-1, active), glucagon, insulin, leptin, monocyte chemo attractant protein 1 (MCP-1), and total pancreatic polypeptide YY (PYY). Assay sensitivities ranged from 0.6-87pg/mL.

### Serum metabolite profiling

Serum metabolites of samples collected from three time-points i.e., baseline (prior to drink consumption), 30 and 120min of OGTT were analyzed and profiled by ^1^H NMR spectroscopy (Bruker Advance 600, Canada) as previously described by our laboratory [16]. Briefly, serum samples (350 μL) were first filtered through pre-washed 10-kDa ultra centrifugal filters, and the filtrate was transferred to phosphate buffer containing NaN_3_ and dimethyl silapentane sulfonate (DSS). Samples were brought to a final volume of 450 μL with D_2_O, so that the concentration of DSS in the sample remained at 0.5M, before the analysis. Resultant data was individually processed and profiled using Chenomx NMR Suite 7.5 (Chenomx, Canada).

### Preliminary SNP analysis

As B-vitamin linked genetic variants regulate flux and elimination, a precursory examination of gene-metabolite interactions was conducted. All participants provided 5mL of saliva in a genetic analysis tube containing a preservative (Genotek, Canada). Comprehensive genotyping for polymorphisms at defined loci was conducted (933 202 SNP Chip, Illumina, OmniExpress Plus Genotyping BeadChip). In order to examine gene-metabolic interactions, the literature was assessed for high global frequency alleles affecting vitamin B metabolism. The goal was a proof of principle inquiry based on high B vitamin intake from the FB rather than a comprehensive genetic analysis or genome wide association study. Our group has previously used this approach with high sensitivity during nutritional challenges [17]. Vitamin B linked one carbon metabolism SNPs examined in this study and their primary associated function are shown in S2 Table and included rs4654748 (ALPL), rs1999594 (MTHFR), rs651852 (BHMT), rs2851391 (CBS), rs1801222 (CUBN) and rs526934 (TCN) [11, 12, 18].

### Statistical analyses

Area-under-the-curve (AUC) was calculated using the trapezoidal method [19]. Shapiro-Wilk confirmed normality, paired-samples t-test was conducted to identify treatment affects (Prism 7.03, GraphPad, USA). Expected genotype frequencies where calculated based on their Global Minor Allele Frequency (GMAF) as previously described [20] and compared with their observed frequencies by a chi-squared test to ensure Hardy-Weinberg equilibrium. A two-way ANOVA was applied to detect gene-metabolite interactions of the serum samples collected from the OGTT. Statistical significance was set at p<0.05.

For metabolomics, multivariate data analyses and model validation were performed in R (version 3.3.2) using “ropls” package [21] as described. Principal component analysis (PCA) and supervised orthogonal partial least square discriminant analysis (OPLS-DA) were applied to evaluate metabolite patterns with treatment. Model validation was performed using a ten-fold cross-validation method [22, 23]. The average ratio of total sum of squared errors (Q2Y) was compared to the percentage of Y variance captured in the total model (R2Y). Metabolites with Variable Importance in Projection (VIP) value more than 1 and raw p value ≤0.05 (independent t-test) were considered to be significant [21].

## Results

### Subjects

A total of twenty healthy adolescents participated in the study, and all completed both arms of the study. Basic anthropometric data is presented on Table 1. None of the participants showed any adverse effects to the consumption of the FB.

**Table 1:** Basic demographic and anthropometric information of participants.

Parameter	Mean (SD)
Age (years)	17 ± 2.2
Gender	10M;10F
BMI (kg/m2)	22.6 ± 4.4
Body fat %	20.3 ± 5.6
Body lean %	76.3 ± 5.5
Waist Circumference (cm)	76.8 ± 10.5
Tanner stage count	4.3 ± 0.8
Fasting blood glucose (mmol/l)	4.5 ± 0.4
Fasting insulin (pmol/l)	89.9 ± 44.9
Blood Pressure Systolic (mmHg)	122.4 ± 16.3
Blood Pressure Diastolic (mmHg)	66.6 ± 9.5

### Laboratory analyses

**Fig 1:**
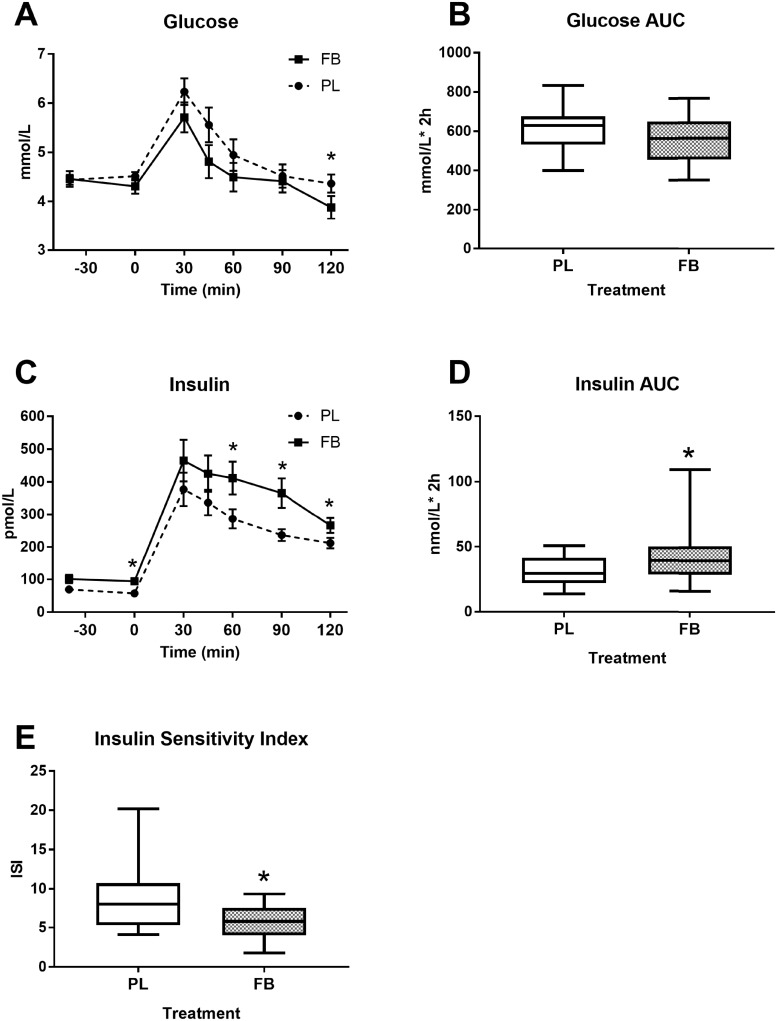
Plasma glucose and insulin excursion in response to an OGTT. Figures show glucose concentration over time (A), glucose AUC (B), insulin concentration over time; (C), insulin AUC (D) and the composite Matsuda index (E). Values represent the means ± SE, n = 20. *Indicates significant difference between PL and FB at p≤0.05.

**Fig 2:**
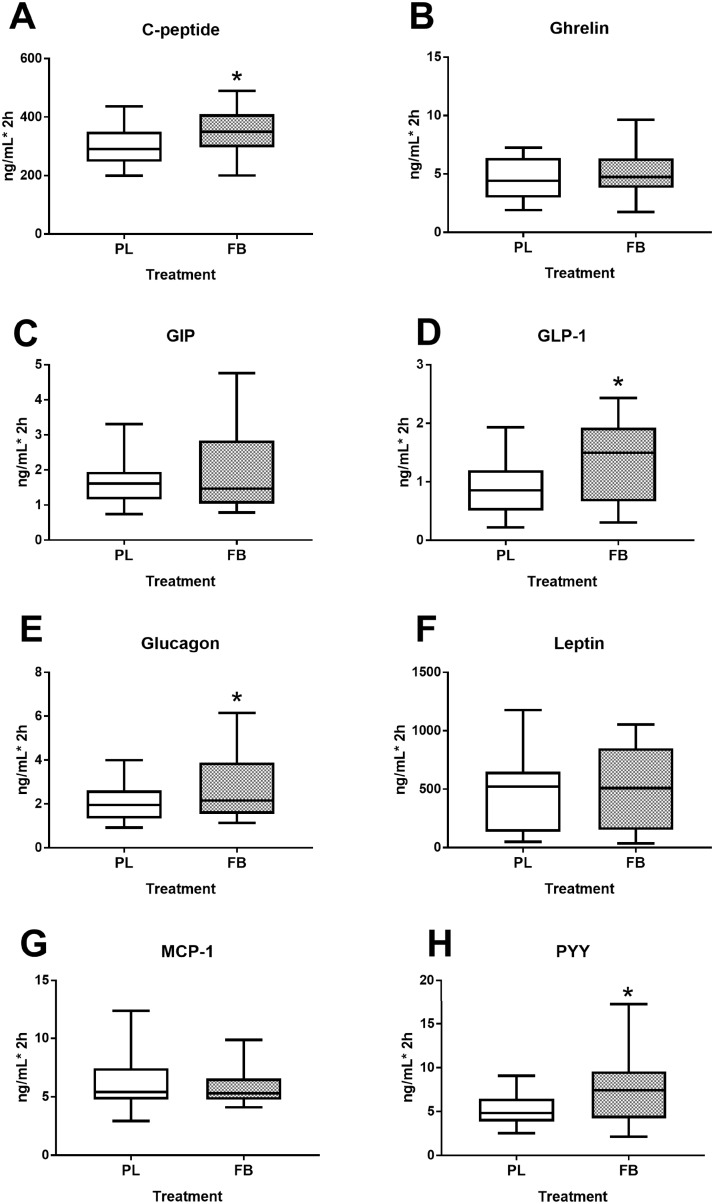
Impact of a FB administration on metabolic hormone responses to an OGTT. Levels reflect AUC for the OGTT (0–120 minutes) for C-Peptide (A), Ghrelin (B), GIP (C), GLP-1 (D), Glucagon (E), Leptin (F), MCP-1 (G), and PYY (H). Values represent the means ± SE, n = 20. *Indicates significant difference between PL and FB at p≤0.05.

Plasma glucose and insulin excursion over time is shown in Fig 1. Concentrations of glucose reached an apogee at 30min and continued to fall thereafter. Even though AUC for glucose (Fig 1B) was not significantly different between the groups (p = 0.111), its concentration tended to be lower in FB group compared to PL group at several time-points (p = 0.012, Fig 1A). On the other hand, plasma insulin AUC (Fig 1D) was significantly (p = 0.003) higher in the FB group compared to PL group. Insulin sensitivity as assessed by the composite Matsuda Insulin Sensitivity Index (ISI) [24] was 28% lower with FB (p = 0.002, Fig 1E). Likewise, AUC of other metabolic hormones including C-peptide (p = 0.017), GLP-1 (p<0.001), glucagon (p = 0.015) and PYY (p = 0.001) were elevated in FB group compared to PL. However, AUC of ghrelin, GIP, leptin and MCP-1 did not differ between the treatment groups (Fig 2).

### Serum metabolite profiling

**Fig 3:**
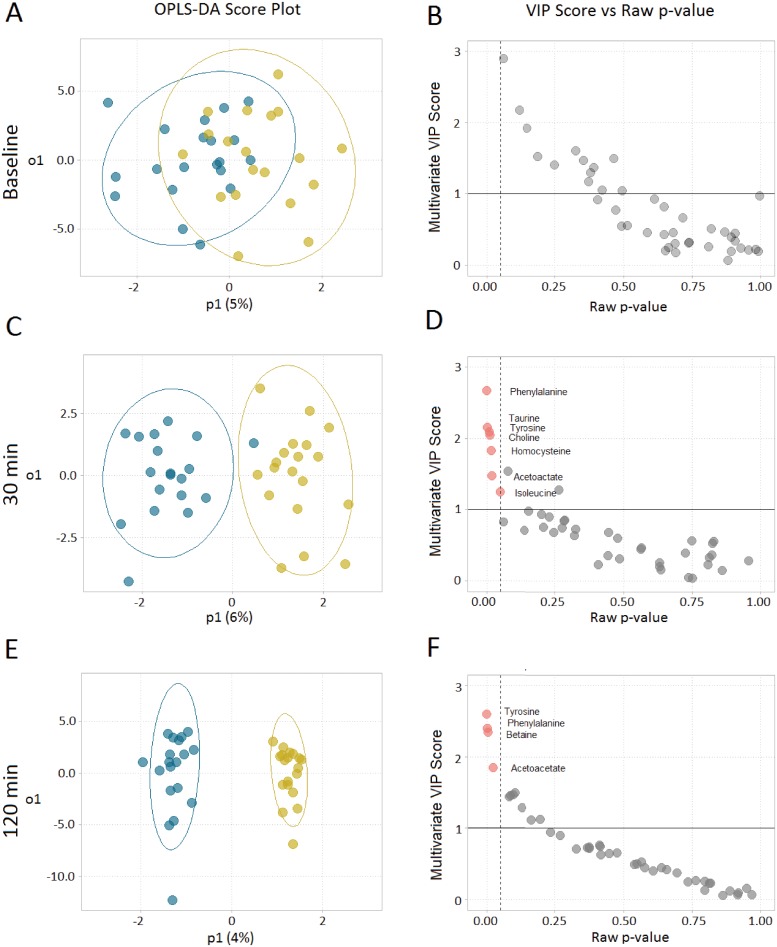
Serum metabolomics analysis in response to FB consumption at 0, 30 and 120min. OPLS-DA scores (A, C, E) and corresponding metabolites (B, D, F), red dots indicate significant metabolites (raw p-value ≤0.05 and VIP > 1), derived from the ^1^H-NMR spectra show progressive separation of treatments over time. n = 20.

Metabolomics analysis was performed on serum samples collected at baseline, 30 min and 120 min of OGTT. A total of 42 metabolites (S3 Table) were detected by ^1^H-NMR spectroscopy. At baseline, there was no separation between treatment groups, and the OPLS-DA model was not significant (p>0.05). However, there was an incremental separation of the two treatment groups over time at 30 and 120min indicating a strong effect of FB on circulating serum metabolites (Fig 3A, 3C and 3E). Cross validation metrics for OPLS-DA models at 30min and 120min were R2Y = 0.84, Q2 = 0.673, p<0.001 and R2Y = 0.972, Q2 = 0.639, p<0.001 respectively. Both models demonstrated separation between treatments (p<0.05) within acceptable limits of both fit and predictive ability (R2 > 0.7, Q2 > 0.5). Significant metabolites identified by our model were: phenylalanine, taurine, tyrosine, choline, homocysteine, acetoacetate and isoleucine for 30min (Fig 3D); and tyrosine, phenylalanine, betaine and acetoacetate for 120min (Fig 3F).

### Precursory gene-metabolite interactions

**Fig 4:**
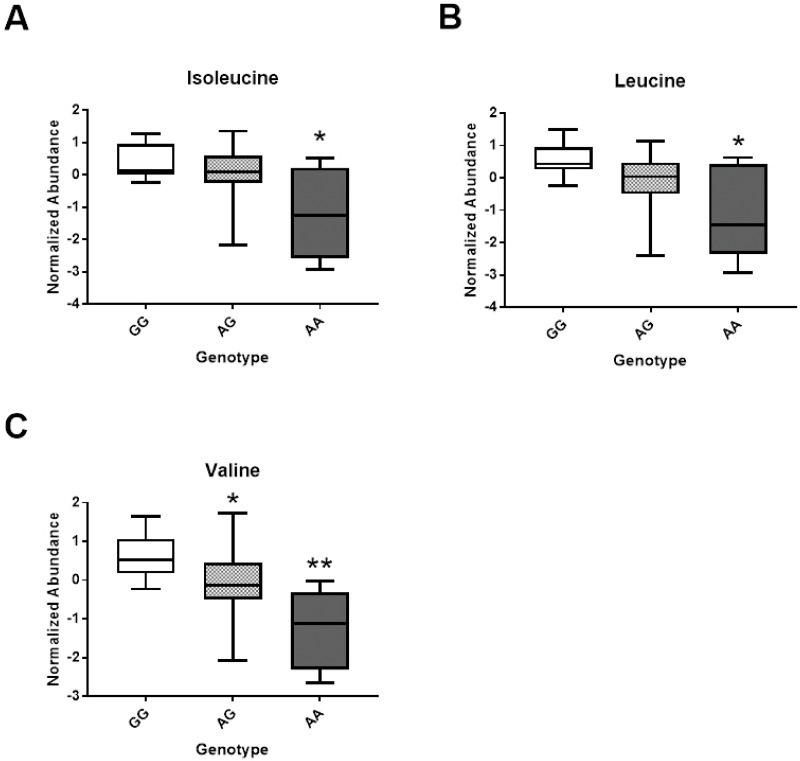
Preliminary analysis of gene-metabolite interactions with FB administration. Box plots for significant main gene effects for rs651852, BHMT polymorphism are shown for Isoleucine (A), Leucine (B) and Valine (C), * or ** represent significant difference (p<0.05) between genotypes.

Although limited by our small sample size, we undertook an exploratory examination of gene-metabolite interactions within our study. Genotype distributions for all selected SNPs were not different from expected, Hardy-Weinberg equilibrium ratios, χ^2^ (p>0.05). Examination of gene-metabolite interactions showed a significant main genetic effect of rs651852, betaine-homocysteine s-methyltransferase (BHMT) for isoleucine, leucine and valine concentrations (Fig 4A–4C) at 120min. Individuals with homozygous A allele in rs651852 had significantly lower serum concentrations of the above branched-chain amino acids compared to their G allele counterparts. All other SNPs examined including rs1801222, rs526934, rs4654748, rs1999594 and rs2851391 exhibited a significant treatment effect only; but did not show either main genetic or interaction effect (S4 Table). These novel findings will require validation in a larger cohort to be confirmed.

## Discussion

Vitamin containing FB are widely consumed by adolescents [7, 8]. This study evaluated the impact of a highly fortified vitamin B containing cocktail on glucose metabolism, metabolic hormone responses and metabolite profiles in adolescents. Major findings of the current study are as follows: (i) the ingested FB disproportionately increased the insulin response to an oral glucose challenge; (ii) the FB differentially affected the secretion of metabolic hormones and (iii) FB transiently altered one carbon, B-vitamin linked amino acid metabolism in response to ingestion. These results suggest that FB could have metabolic impacts that are ingredient and concentration dependent.

To decipher the impact of an FB on glucose and insulin responses, adolescent subjects consumed PL or FB followed 40 minutes later by a standard OGTT. This time was chosen as it allowed for full absorption of the FB and its subsequent impacts on glucose tolerance to be realized. Results showed no difference in glucose levels with FB consumption, both glucose peak and total area under the curve during the OGTT remained unchanged between treatments. However, examination of insulin levels revealed a 24% increase over the OGTT along with significantly elevated C-peptide levels with FB consumption. These responses resulted in a 28% decline in the composite ISI, an indicator of glucose disposal relative to insulin levels. The FB employed in the present study, 5h Energy Decaffeinated, contained several insulinogenic ingredients including sucralose and free amino acids [25, 26]. Higher insulin concentrations without altered glycemia with acute sucralose consumption has been previously reported [27]. Likewise, there is a growing body of evidence showing that the consumption of artificial sweeteners may disrupt glucose homeostasis and is positively associated with host metabolic derangements including obesity, diabetes and cardiovascular disease with chronic consumption [26, 28, 29].

Next, we examined the influence of FB ingestion on gastrointestinal hormone profiles in response to the OGTT. The incretins GLP-1 and GIP are gut-derived hormones that potentiate postprandial insulin secretion [30] and are necessary for normal glucose tolerance [31]. In the present study, we show significantly higher levels of GLP-1, PYY and glucagon with a glucose load with FB pretreatment compared to the PL group. The observed increases in PYY levels with FB consumption were most likely a result of the high levels of free amino acids in the FB including tyrosine and phenylalanine [32]. Augmented GLP-1 secretion with FB may have also resulted from the sucralose contained in the beverage. This finding is consistent with previous observations showing diet soda consumption (containing artificial sweeteners) to elevate GLP-1 secretion in healthy adolescents [33].

Common in many FB, our test beverage also contained levels of B vitamins (B_6_, folic acid and B_12_) at concentrations above recommended levels. Of note, the Recommended Dietary Allowance (RDA) for vitamin B_6_ and B_12_ range from 1.0–1.3mg/d and 1.8–2.4μg/d for children aged 9-18y respectively [3]. The test beverage contained 40mg of vitamin B_6_ and 500μg of vitamin B_12_ which are 3000% and 20000% of the RDA (S1 Table). Perhaps more concerning is this proximity to its upper limit (UL) of ingredients with either a single serving or multiple servings per day. For example, a single serving is below the B_6_ UL of 60mg per day for 9-13y, but more than one serving would exceed this limit. Although B-vitamins play crucial roles in numerous enzymatic reactions, high doses have been associated with toxicity [34, 35]. Whilst toxicity is unlikely due to high tolerance limits [34], several servings of FB might be a concern to young children and adolescents due to their lower body mass.

**Fig 5:**
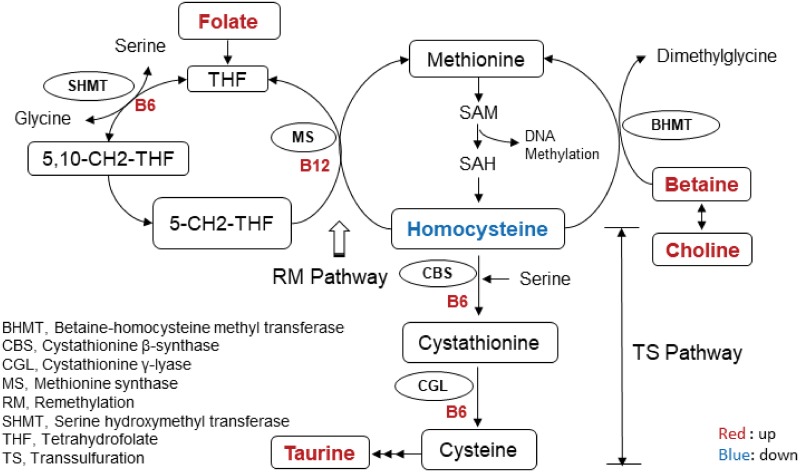
Schematic of the pathways and metabolites in B-vitamin-linked one carbon metabolism affected by FB consumption in adolescents. Affected metabolites are highlighted. Red indicates upregulation while blue indicates a downregulation with FB administration.

To gain insight into the metabolic alterations resulting from FB ingestion, a metabolomics approach was employed. Results demonstrated no difference in serum metabolites at baseline between the two treatments. However, at both early (30 min) and late time points (120 min), a progressive separation of profiles was observed, reflecting absorption and distribution of FB ingredients throughout the circulation. Ingested FB components appearing in the metabolomics analysis included taurine, phenylalanine, tyrosine and choline (administered as citicoline). Unexpectedly, differences in a number of other metabolites that were not ingested as part of the FB were also observed between treatments including homocysteine, betaine and acetoacetate. These differences implicate shifts in the pathways governing these metabolites including vitamin B-linked one carbon metabolism with FB ingestion. Notably, we show homocysteine levels to be reduced 30min after the glucose challenge with the FB. This is likely due to an increase in clearance, from activation of the B-vitamin-dependent enzymes linked to sulfur amino acid metabolism either through the remethylation or transsulfuration pathways (Fig 5). Additionally, choline (an ingredient in FB) is converted to betaine (p<0.05 at 120min) resulting in further augmentation of homocysteine clearance through the BHMT enzyme system. Similar alterations and reduced homocysteine levels have been observed with supplementation of both betaine and choline in the diet [36, 37]. Atkinson *et al*.[36] observed a significant decrease in plasma total homocysteine concentrations of healthy men 4h after consuming a betaine and choline-rich meal, supporting our finding that the FB consumed in this study likely resulted in alterations to pathways governing vitamin B-linked one carbon metabolism.

Upon examination of metabolite profiles with FB, we noted some variable patterns that led us to examine the influence of genetic variation for enzymes related to vitamin B-metabolism. Our laboratory has found that the contribution of genetic variation to metabolic flux can often be revealed with a targeted nutritional challenge (FB administration in this case) [17]. Our preliminary analysis revealed one SNP to significantly interact with metabolite profiles in response to the FB challenge, rs651852 (BHMT). Described as a central ‘short cut’ through the methylation cycle, this enzyme is central to converting homocysteine to methionine which donates a methyl group to DNA, proteins, lipids, and other intracellular metabolites [38]. In the present study, this one carbon metabolism gene polymorphism significantly influenced serum isoleucine, leucine and valine concentrations, with the minor allele resulting in lower concentrations at 120min post FB ingestion. This novel finding indicates that genetic variance likely alters the metabolic responsiveness to FB consumption, although this needs to be confirmed in a larger cohort.

While this study shows FB to have metabolic impacts in adolescents, it must be emphasized that our FB was administered as a commercially available mixed ingredient product. As such, it is not possible to pinpoint specific responses to a single nutrient, and the observed responses are likely product and ingredient specific. From a clinical standpoint, the finding related to the impact of FB on glucose tolerance warrants further investigation. A 28% reduction in the ISI in healthy adolescents following FB consumption could be exaggerated in overweight and obese adolescents. There may also be consequences for healthy eating. The greater the intake of discretionarily fortified foods and beverages, the lower the intake of fruits and vegetables, milk products, meats and alternatives suggesting the replacement of healthy eating practices with convenience foods and beverages such as vitamin loaded FB [39].

In conclusion, this study identified metabolic impacts of a highly fortified vitamin beverage on glucose metabolism, metabolic hormones and serum metabolites in adolescents. Although these impacts are likely short-lived, results show that beverages fortified with high amounts of vitamins are not metabolically inert, but likely result in greater insulin secretion, differential hormone secretion and elevated one-carbon flux to process the excessive vitamin loads. Given this, ongoing evaluation of products with levels of fortification of vitamins and minerals that exceed the Recommended Dietary Allowance appears warranted.

## Supporting information

S1 Table Ingredient list present in fortified beverage (FB) and their percent daily value. (EPS)

S2 Table Selected genes and their SNPs linked to B-vitamin metabolism with their significant levels. (EPS)

S3 Table List of compounds detected by ^1^H- NMR spectroscopy from samples collected at baseline, 30 min and 120 min of OGTT with their corresponding ppm used for identification. (EPS)

S4 Table Effect of selected SNPs on serum metabolites showing their 2-way ANOVA p-values (FDR corrected) of gene, treatment and interaction effects. (EPS)

S1 Fig An overview of the study design and time-points where blood samples are collected. Treatments are given at baseline which is -40 min before the OGGT. Blood samples collected from all time-points starting from 0 min till 120 min were used for quantifying different analytes including glucose, insulin, and gut hormones. The samples collected at baseline, 30 min and 120 min were used for metabolomics. FB, Functional beverage.
(EPS)
